# Macrophage activation syndrome and pulmonary arterial hypertension in a patient with adult-onset Still disease: A case report

**DOI:** 10.1097/MD.0000000000017427

**Published:** 2019-11-01

**Authors:** Furong Zeng, Guangtong Deng, Hui Luo, Xiaoxia Zuo, Yanli Xie

**Affiliations:** a Department of Rheumatology and Immunology, Xiangya Hospital, The Institution of Rheumatology and Immunology; b Xiangya School of Medicine, Central South University, Changsha, Hunan, China.

**Keywords:** Adult-onset Still disease, macrophage activation syndrome, pulmonary arterial hypertension

## Abstract

**Introduction:**

Macrophage activation syndrome (MAS) and pulmonary arterial hypertension (PAH) are rare and life-threatening complications of adult-onset Still disease (AOSD).

**Patient concerns:**

We reported an interesting case of a 25-year-old AOSD patient with MAS and PAH, and the patient was found to have right anomalous pulmonary venous connection accompanied by an atrial septal defect.

**Diagnosis:**

MAS was diagnosed as a complication of AOSD. PAH was contributed probably by right anomalous pulmonary venous connection.

**Interventions:**

The patient dramatically improved with methylprednisolone (80 mg I.V. daily) plus supportive treatments, without interleukin (IL) inhibitors or ciclosporin A given.

**Outcomes:**

The patient's serum hepatic enzyme levels dropped and hemocytes rose within 1 week.

**Conclusion:**

Other causes need to be excluded carefully before giving a diagnosis of PAH with AOSD. Early diagnosis and aggressive treatments are pivotal to improve the quality of life and the survival of patients.

## 1 Introduction

Adult-onset Still disease (AOSD) is a rare systemic inflammatory disorder of unknown etiology, with an incidence of 1 to 4 cases per million.^[1]^ It is characterized by recurrent fevers, arthralgia, leukocytosis, and maculopapular rash.^[2]^ Macrophage activation syndrome (MAS) is a life-threatening complication of AOSD and its incidence is higher in AOSD than other rheumatic diseases. MAS is characterized by an unregulated immune response with hyper-expansion of CD8+ T cells and uncontrolled macrophage activation.^[3]^ MAS shares similar clinical and pathophysiological features with AOSD such as high fever, splenomegaly, hyperferritinemia, and excessive natural killer (NK) cell and CD8+ T cell activity.^[4]^ Pulmonary arterial hypertension (PAH) is a disease caused by vasoconstriction and vascular remodeling, and manifest with pulmonary artery resistance elevation and high pressure. PAH has been reported to be associated with several connective tissue diseases (CTDs), such as systemic sclerosis, systemic lupus erythematosus, and mixed CTD, but rare in AOSD.^[5]^ Here we described a rare case of AOSD accompanied by MAS and PAH, and image study revealed right anomalous pulmonary venous connection accompanied by an atrial septal defect.

## 2 Case presentation

A 25-year-old woman was admitted to rheumatology and immunology department because of recurrent joint pain for 2 years, fever, and rash for 20 days. The arthralgia appeared and localized at her bilateral knees, wrists, elbows, shoulders, and hips. Twenty days before admission, she began to experience high fever (>39 °C) with associated rash.

The patient didn’t have medical, family, or psychosocial history. Laboratory data revealed a high white blood cell count (WBC 14.7 × 10^9^/L, with 87.1% neutrophils), slightly decreased red blood cell count (RBC 3.67 × 10^12^/L) and hemoglobin level (Hb 110 g/L), but elevated C-reactive protein level (CRP 99.3 mg/L, reference <8 mg/L) and erythrocyte sedimentation rate (ESR 114 mm/h, reference <26 mm/h), as well as elevated serum hepatic enzyme levels (aspartate transaminase [AST] 51.7 U/L, reference ≤35 U/L); alanine transaminase (ALT 56.2 U/L, reference ≤40 U/L), lactate dehydrogenase (LDH 560 U/L, reference 120–250 U/L), and serum interleukin (IL)-6 (43.6 pg/mL; reference <5.9 pg/mL) level. Serum immunoglobulin, IgG, IgA, and IgM levels were normal, antinuclear antibodies, and rheumatoid factors were negative. All specific cultures and serology antibodies for infection detection were negative as well. Abdominal ultrasound indicated splenomegaly. A bone marrow biopsy was negative for MAS. Therefore, AOSD was proposed on admission according to Yamaguchi criteria (with a sensitivity of 96.2% and a specificity of 92.1%).^[6]^

The patient received supportive treatments including liver protection drugs and nutrition support without glucocorticoid treatment immediately in case of infection. But 3 days later, persistent fever of 40 °C, abdominal pain, and vomit occurred. Laboratory tests again showed obvious decline of WBC count (5.1 × 10^9^/L), Hb (98 g/L), and platelet count (35 × 10^9^/L) in comparison with the previous data on admission. Meanwhile, low NK cell activity (3.15%), elevated triglyceride (TG) levels (179 mg/dL), increased serum sIL-2R level (3310 U/mL), and elevated serum ferritin level (33,405 ng/mL, reference 13–150 ng/mL) were presented as well. AST (204.7 U/L) and ALT (142.7 U/L) levels has raised further. At this time, this patient's presentation fulfilled HLH-2004 criteria for diagnosing MAS.^[7]^ Therefore, this patient was given methylprednisolone 80 mg I.V. daily plus supportive treatments. The patient's serum hepatic enzyme levels dropped and hemocytes rose within 1 week.

**Figure 1:**
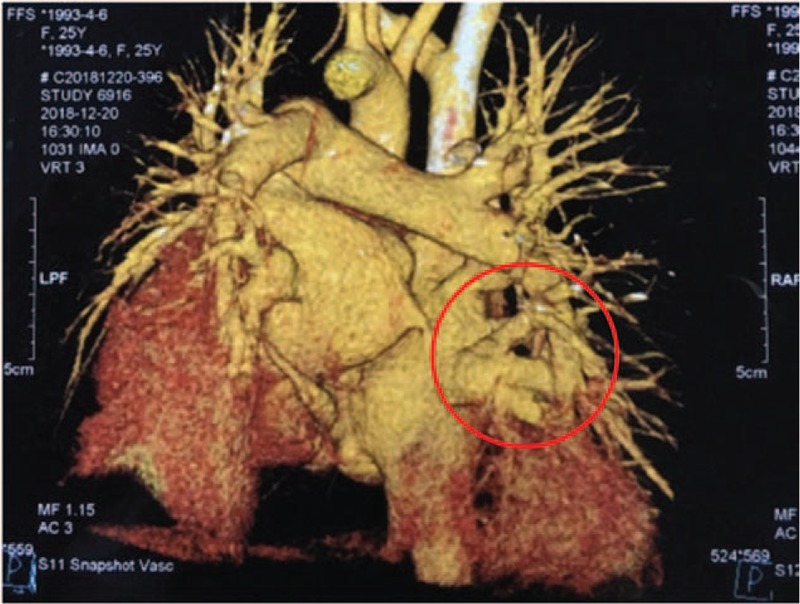
CTA shows right superior pulmonary vein, right middle vein, and right inferior pulmonary vein went into right atrium directly. CTA = computed tomography angiography.

**Figure 2:**
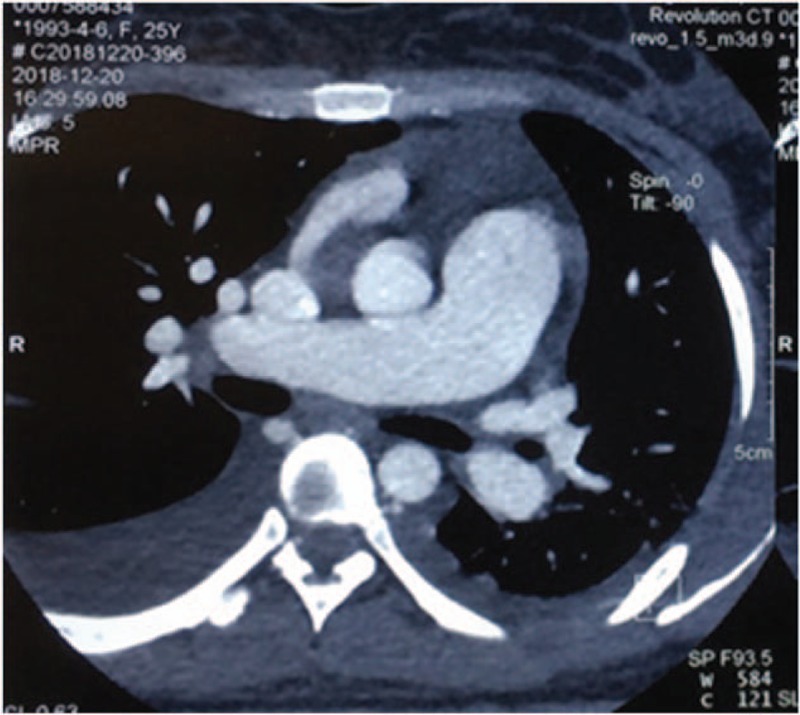
Computed tomography chest with contrast, axial cut shows markedly dilated pulmonary trunk 29 mm.

Interestingly, both her chest computed tomography (CT) and echocardiography revealed enlarged right atrium and ventricle, thickened pulmonary trunk, and PAH (estimated pulmonary arterial pressure: 76 mmHg). However, this patient didn’t have shortness of breath or dyspnea. PAH is a rare complication of AOSD, with an overall prevalence of 4.8%.^[8]^ So we tried to find whether there are other causes co-existed in this patient except for the common etiologies such as infections, malignancies, or rheumatic diseases inducing her PAH. To our surprise, computed tomography angiography (CTA) revealed right superior pulmonary vein, right middle vein, and right inferior pulmonary vein entered into patient's right atrium directly (Fig. 1), a pulmonary artery enlarged to 29 mm (Fig. 2) and a 5 mm atrial septal defect were detected in this patient as well.

This study was approved by the Ethics Committee of Xiangya Hospital of Central South University, Changsha, Hunan, China. Informed written consent was obtained from the patient for publication of this case report and accompanying images.

## 3 Discussion

MAS is a life threatening complication of AOSD with high mortality rate ranging from 10% to 20%.^[3,9]^ It's rare, but not uncommon in AOSD, with an incidence of 12% to 14% in AOSD cases.^[4]^ The most common triggers for MAS during AOSD course are AOSD flare ups, comorbid with infections, and medications in use.^[10]^ MAS patients with apparent triggering factors showed lower death rates than those without.^[10]^ The pathophysiological mechanisms of AOSD are not well understood. It has been proposed that the defect of NK and CD8+ T cells in granule mediated cytotoxicity^[11]^ and the altered homoeostasis of dendritic cells due to dysfunction of perforin and Fas/FasL mediated pathway,^[12]^ lead to enhanced antigen presentation and repetitive interferon (INF)-γ-dependent stimulation of toll-like receptors, and followed by uncontrolled activation of macrophages, histiocytes, and T cells are the underline mechanism. This chain of activation produces a cytokine storm due to overproduction of pro-inflammatory cytokines such as INF-γ, tumor necrosis factor-α (TNF-α), and ILs, which are related to the development of the main clinical and laboratory features and contribute to tissue damage and progressive systemic organ failure.^[3]^

Hyperferritinemia is a common feature shared by both AOSD and MAS.^[13]^ It has been reported that it is not only a diagnostic marker of AOSD,^[14]^ but also a prognostic marker of MAS.^[9]^ It independently associated with severity of the clinical manifestations of AOSD^[15]^ and MAS-related death.^[16]^ Ferritin is not only considered as a consequence of the inflammation but also thought to be involved in pathogenic mechanisms.^[13,17]^ The inflammatory cytokines stimulate macrophages to release H-ferritin, the heavy subunit of ferritin, via Fer2 activation. The increased H-ferritin further enhance pro-inflammatory cytokines release, thus perpetuating the inflammatory status.^[18,19]^ The pro-inflammatory cytokines produced during AOSD, mainly IL-18, may play a major role in MAS initiation and may also predict the development of MAS in AOSD. Those cytokines can impair NK cell cytotoxicity and trigger Th1 response which inducing IFN-γ secretion, a possible pathogenic process in patients experiencing the complication.^[20]^ High-dose glucocorticoid, even pulse dose (500–1000 mg/daily for a maximum of 6 cycles) are recommended to treat patients with MAS, and then followed by IL-1 or IL-6 inhibitors. If refractory, etoposide or ciclosporin A are recommended.^[4,21]^

This patient was found to present with PAH without clinical manifestation. We at first thought it was contributed by AOSD. There were few similar cases reported in the past 5 years.^[5,22–28]^ PAH is a rare and very severe AOSD manifestation, but the prevalence is probably under estimated because only symptomatic cases were reported. PAH may occur at AOSD onset or later course and seems to mainly affect women.^[24]^ Rapidly progressing dyspnea is the main clinical presentation, followed by the signs of right atrial hypertrophy. Echocardiography is useful for screening of PAH by measuring the systolic pulmonary artery pressure (>35 mmHg) and left ventricle ejection fraction.^[4,14]^

The exact pathophysiology of PAH is not clearly understood. Immune system dysregulation is reported to play a pivotal role in the pathogenesis and progression of PAH due to actively recruiting inflammatory cells and irreversibly remodeling the pulmonary vasculature, followed by overproduction of pro-inflammatory cytokines, including IL-1β, IL-18, IL-6, IL-18, and TNF-α.^[4,8]^ PAH is the result of dysfunctional interaction between the immune system and the pulmonary endothelium. Pulmonary microangiopathy may also participated in pathogenesis of PAH.^[29]^

Interestingly, in our case, the patient was found presenting with right anomalous pulmonary venous connection accompanied by a 5 mm atrial septal defect. Anomalous pulmonary venous connection is a rare cardiovascular abnormality caused by dysplasia of embryo. Pulmonary venous return is obstructed due to overload of right atrium, leading to PAH and eventually right heart failure. In this patient, the PAH is probably contributed by right anomalous pulmonary venous connection, but the contribution of AOSD cannot be excluded. The patient doesn’t have any clinical manifestation because the atrial septal defect relieves symptoms of PAH in some degree. Unfortunately, she refused to do the surgery because of financial burden. We suggested her to visit the cardiologist regularly.

In summary, we reported a rare case of a 25-year-old woman with AOSD as well as congenital heart disease, complicated by MAS and PAH. Early screening of PAH in AOSD patients with echocardiography is necessary. When giving a diagnosis of PAH with AOSD, we should rule out other causes inducing PAH especially some cardiac diseases hardly revealed by regular test. Additionally, only corticosteroids and supportive treatments were used at the very beginning stage of MAS, and IL inhibitors or ciclosporin A were not applied in our case, emphasizing the importance of maintaining a high clinical suspicion for MAS and initiating treatment early.

## Author contributions

**Methodology:** Hui Luo.

**Resources:** Guangtong Deng.

**Supervision:** Xiaoxia Zuo.

**Writing – original draft:** Furong Zeng.

**Writing – review & editing:** Yanli Xie.

## References

[1] Castaneda S Blanco R Gonzalez-Gay MA Adult-onset Still's disease: advances in the treatment. Best practice & research. Clin Rheumatol 2016;30:222–38. 10.1016/j.berh.2016.08.003 27886796

[2] Efthimiou P Kadavath S Mehta B Life-threatening complications of adult-onset Still's disease. Clin Rheumatol 2014;33:305–14. 24435354 10.1007/s10067-014-2487-4 PMC7102228

[3] Ramos-Casals M Brito-Zeron P Lopez-Guillermo A Adult haemophagocytic syndrome. Lancet 2014;383:1503–16. 24290661 10.1016/S0140-6736(13)61048-X

[4] Mitrovic S Fautrel B Complications of adult-onset Still's disease and their management. Expert Rev Clin Immunol 2018;14:351–65. 29658384 10.1080/1744666X.2018.1465821

[5] Mehta MV Manson DK Horn EM An atypical presentation of adult-onset Still's disease complicated by pulmonary hypertension and macrophage activation syndrome treated with immunosuppression: a case-based review of the literature. Pulm Circ 2016;6:136–42. 27162622 10.1086/685112 PMC4860549

[6] Yamaguchi M Ohta A Tsunematsu T Preliminary criteria for classification of adult Still's disease. J Rheumatol 1992;19:424–30. 1578458

[7] Henter JI Horne A Arico M HLH-2004: diagnostic and therapeutic guidelines for hemophagocytic lymphohistiocytosis. Pediatr Blood Cancer 2007;48:124–31. 16937360 10.1002/pbc.21039

[8] Narvaez J Mora-Liminana M Ros I Pulmonary arterial hypertension in adult-onset Still's disease: a case series and systematic review of the literature. Semin Arthritis Rheum 2019;49:162–70. 30580885 10.1016/j.semarthrit.2018.11.007

[9] Ruscitti P Rago C Breda L Macrophage activation syndrome in Still's disease: analysis of clinical characteristics and survival in paediatric and adult patients. Clin Rheumatol 2017;36:2839–45. 28914368 10.1007/s10067-017-3830-3

[10] Bae CB Jung JY Kim HA Reactive hemophagocytic syndrome in adult-onset Still disease: clinical features, predictive factors, and prognosis in 21 patients. Medicine (Baltimore) 2015;94:e451. 25634183 10.1097/MD.0000000000000451 PMC4602979

[11] Arceci RJ When T cells and macrophages do not talk: the hemophagocytic syndromes. Curr Opin Hematol 2008;15:359–67. 18536575 10.1097/MOH.0b013e3282f97f88

[12] Chen M Felix K Wang J Critical role for perforin and Fas-dependent killing of dendritic cells in the control of inflammation. Blood 2012;119:127–36. 22042696 10.1182/blood-2011-06-363994 PMC3251225

[13] Rosario C Zandman-Goddard G Meyron-Holtz EG The hyperferritinemic syndrome: macrophage activation syndrome, Still's disease, septic shock and catastrophic antiphospholipid syndrome. BMC Med 2013;11:185. 23968282 10.1186/1741-7015-11-185 PMC3751883

[14] Feist E Mitrovic S Fautrel B Mechanisms, biomarkers and targets for adult-onset Still's disease. Nat Rev Rheumatol 2018;14:603–18. 30218025 10.1038/s41584-018-0081-x PMC7097309

[15] Gerfaud-Valentin M Maucort-Boulch D Hot A Adult-onset still disease: manifestations, treatment, outcome, and prognostic factors in 57 patients. Medicine (Baltimore) 2014;93:91–9. 24646465 10.1097/MD.0000000000000021 PMC4616309

[16] Ruscitti P Cipriani P Ciccia F Prognostic factors of macrophage activation syndrome, at the time of diagnosis, in adult patients affected by autoimmune disease: analysis of 41 cases collected in 2 rheumatologic centers. Autoimmun Rev 2017;16:16–21. 27664384 10.1016/j.autrev.2016.09.016

[17] Ruscitti P Cipriani P Di Benedetto P Increased level of H-ferritin and its imbalance with L-ferritin, in bone marrow and liver of patients with adult onset Still's disease, developing macrophage activation syndrome, correlate with the severity of the disease. Autoimmun Rev 2015;14:429–37. 25599955 10.1016/j.autrev.2015.01.004

[18] Ruscitti P Ciccia F Cipriani P The CD68(+)/H-ferritin(+) cells colonize the lymph nodes of the patients with adult onset Still's disease and are associated with increased extracellular level of H-ferritin in the same tissue: correlation with disease severity and implication for pathogenesis. Clin Exp Immunol 2016;183:397–404. 26540556 10.1111/cei.12738 PMC4750598

[19] Ruscitti P Cipriani P Ciccia F H-ferritin and CD68(+) /H-ferritin(+) monocytes/macrophages are increased in the skin of adult-onset Still's disease patients and correlate with the multi-visceral involvement of the disease. Clin Exp Immunol 2016;186:30–8. 27317930 10.1111/cei.12826 PMC5011370

[20] Giacomelli R Ruscitti P Shoenfeld Y A comprehensive review on adult onset Still's disease. J Autoimmun 2018;93:24–36. 30077425 10.1016/j.jaut.2018.07.018

[21] Schulert GS Grom AA Macrophage activation syndrome and cytokine-directed therapies. Best Pract Res Clin Rheumatol 2014;28:277–92. 24974063 10.1016/j.berh.2014.03.002 PMC4074772

[22] Guilleminault L Laurent S Foucher A Pulmonary arterial hypertension in adult onset Still's disease: a case report of a severe complication. BMC Pulm Med 2016;16:72. 27160441 10.1186/s12890-016-0237-x PMC4862120

[23] Kadavath S Zapantis E Zolty R A novel therapeutic approach in pulmonary arterial hypertension as a complication of adult-onset Still's disease: targeting IL-6. Int J Rheum Dis 2014;17:336–40. 24581387 10.1111/1756-185X.12324

[24] Lowther GH Chertoff J Cope J Pulmonary arterial hypertension and acute respiratory distress syndrome in a patient with adult-onset stills disease. Pulm Circ 2017;7:797–802. 29168664 10.1177/2045893217712710 PMC5703120

[25] Padilla-Ibarra J Sanchez-Ortiz A Sandoval-Castro C Rituximab treatment for pulmonary arterial hypertension in adult-onset Still's disease. Clin Exp Rheumatol 2013;31:657–8. 23622421

[26] Sinha A Patti R Ambesh P Severe pulmonary hypertension due to adult-onset Still's disease. J Investig Med High Impact Case Rep 2018;6:2324709618757260. 10.1177/2324709618757260 PMC5815417 29468168

[27] Weatherald J Lategan J Helmersen D Pulmonary arterial hypertension secondary to adult-onset Still's disease: response to cyclosporine and sildenafil over 15 years of follow-up. Respir Med Case Rep 2016;19:27–30. 27408785 10.1016/j.rmcr.2016.06.007 PMC4927635

[28] Wong KL Ytterberg SR Frantz RP Resolution of severe pulmonary arterial hypertension complicating adult-onset Still's disease. J Heart Lung Transplant 2016;35:1140–4. 27216503 10.1016/j.healun.2016.03.001

[29] Thakare M Habibi S Agrawal S Pulmonary arterial hypertension complicating adult-onset Still's disease. Clin Rheumatol 2013;32 suppl:S1–2. 19669855 10.1007/s10067-009-1230-z

